# Factors affecting patients’ knowledge about dispensed medicines: A Qualitative study of healthcare professionals and patients in Pakistan

**DOI:** 10.1371/journal.pone.0197482

**Published:** 2018-06-01

**Authors:** Anum Saqib, Muhammad Atif, Raazeyah Ikram, Fatima Riaz, Muhammad Abubakar, Shane Scahill

**Affiliations:** 1 Department of Pharmacy, The Islamia University of Bahawalpur, Bahawalpur, Punjab, Pakistan; 2 School of Management, Massey University, Auckland, New Zealand; Brown University, UNITED STATES

## Abstract

**Background:**

Patients’ knowledge about their prescribed medicines is one of the most important antecedents of successful therapy. Poor knowledge about medicines can lead to serious consequences such as non-adherence and misunderstanding of the significance of adverse events. The objective of this study is to understand the factors that are responsible for a patients’ lack of knowledge regarding their medicines, by taking the perspective of the patient as well as that of healthcare professionals. Much of the work in this area has been undertaken in the setting of developed or semi-developed countries, and there is a scarcity of information from developing nations such as Pakistan.

**Methods:**

This was a large qualitative study set in the hospital outpatient environment in a teaching hospital in the Punjab province of Pakistan. Data were collected from dialogue with patients (n = 19) and healthcare providers (n = 16) i.e., doctors and dispensers (where a dispenser is a person who merely dispenses medicines; i.e. is not a pharmacist) through in-depth semi-structured interviews. Patients having limited knowledge about their dispensed medicines were assessed using a checklist. The healthcare providers were recruited through a convenience sampling strategy, based on their availability and willingness to participate in the study. Based on the objectives of the study, a pilot tested interview protocol was developed, and used to conduct the interviews. The sample size was controlled by using saturation point criteria. All interviews were audio recorded and transcribed verbatim. The data were analyzed to draw conclusions using inductive thematic content analysis.

**Results:**

The analysis of data yielded 31 categories (patients = 19, healthcare professionals = 12), 10 subthemes and three themes. The major themes were healthcare professional-related factors, patient-related factors and system-related factors. The health professional related subthemes included: behaviour and attitude and professional liabilities and liaison. The patient related subthemes included: eagerness of the patients and lack of understanding and misconception. The system-related factors included: patients with special needs, perceived role of the pharmacist, prescription and medicines, and staff workload.

**Conclusion:**

Healthcare professional related, patient related and system related factors have a significant influence on patients’ knowledge about dispensed medicines. The non-professional behaviour of doctors, increased staff workload, inadequate time and attention provided by healthcare professionals to patients, illiteracy of patients, lack of specialized labelling on medicines for illiterate patients and absence of pharmacists at the hospital, were the major concerns identified in this study. The study points to a need for appropriate patient education and counselling with regards medicines, improved coordination between hospital staff, and provision of some basic system-related facilities which are pivotal for enhancing patients’ knowledge and adherence to their treatment regimens.

## Introduction

Most studies on patients’ knowledge of dispensed medicines are from the developed world and very few are from developing Asian countries [1]. One of the essential prerequisites for the optimal use of medicines is sound patient knowledge about prescribed medications [2]. According to the standard medicine use indicators set by the World Health Organization (WHO), the patients’ knowledge of their treatment regimen is an important patient care indicator [3]. In the evaluation of patients’ knowledge of medicines, the following are regarded as essential parameters for safe and effective use; the names of the medicines, the purpose of therapy, the duration of therapy, the dose and frequency of administration and important side effects [4]. Inadequate knowledge of medicines by patients may result in their incorrect use, which can lead to treatment failure and puts the health of the patient at risk. Moreover, lack of knowledge may cause unintended overdose or non-adherence with medicine regimens, resulting in poor outcomes [5]. Undoubtedly, the appropriate healthcare professionals are responsible for educating patients about their medicines, but there are certain limitations to this including; being overburdened, inadequate knowledge of the doctors, patients’ low health literacy levels and a lack of patient interest in learning about the dispensed medicines—which challenges the ease of educating patients.

Multiple studies have demonstrated patients’ poor knowledge of their dispensed medicines [6, 7]. A study conducted in Portugal demonstrated that eight out of 10 patients did not know anything about the medicines prescribed for them [8]. In a similar context, studies from the African continent and in particular Ghana and rural Gambia highlight the importance of patient knowledge of medicines as an antecedent to adherence to medication regimes. Marfo and colleagues [9] evaluated the quality of labelling of medicines in community pharmacy in Ghana to find the duration of therapy and route of administration was not frequently written. Labelling was rated just above average. Likewise, in Botswana Boonstra and co-workers [2] looked at labelling and knowledge of dispensed drugs as quality indicators in primary care and found that patients had a reasonable knowledge of medicines but that family welfare educators did not. In Ethiopia Hirko and Edessa [10] explored the exit knowledge of patients at an outpatient pharmacy from a university hospital. From 422 respondents, they found that the majority of patients poorly understood the name of the dispensed medication, side effects and what to do with missed doses.

In a country like Pakistan, where almost half of the population is illiterate [11] and over two thirds (67%) of the population reside in rural areas [12], lack of education and awareness about medicines is a potential contributor towards poor patient knowledge about prescribed medicines. In the Pakistani context physicians usually diagnose, prescribe and provide medicines-related information for patients. However, doctors seldom educate the patients about appropriate use of their medicines due to their workload and do not give sufficient consultation time to the patients [13, 14]. Pharmacists in Pakistan are mainly appointed to roles as hospital pharmacists in public sector hospitals. Here the major responsibility is the sourcing, storage and distribution of medicine and pharmacists’ clinical roles are minimal. The main responsibilities of the nurse are to administer medicine and provide medicines-related information to inpatients. The majority of medicines available through pharmacies and medical stores are “dispensed” (given out) by personnel who have minimal formal pharmacy education and lack the extent of medicines-related knowledge that a pharmacist has to do the job optimally [15].

The objective of this study is to identify the factors which affect patients’ knowledge about their dispensed medicines. To the best of our knowledge, this is the first qualitative study undertaken in Pakistan which explores patients’ poor knowledge about their dispensed medicines and the underlying reasons for this lack of knowledge. This study is expected to inform policy development and provide a lever for policy initiation, with a view to improving the healthcare system and helping to alleviate disease burden in developing countries such as Pakistan.

## Methods

### Study design, setting and sampling frame

A qualitative study design was employed in which face to face interviews were conducted with patients and healthcare professionals i.e., doctors and dispensers (where a dispenser is a person who merely dispenses the medicines, not a pharmacist who also provides clinical advice) using semi-structured interview schemas.

### Study setting and sampling frame

The sampling frame involved patients and healthcare professionals (including doctors and dispensers) and the study was conducted at the Bahawal Victoria Hospital (BVH), which is situated in the district of Bahawalpur, Punjab province of Pakistan. BVH is a teaching hospital of the Quaid-e-Azam Medical College and was established in 1906. This tertiary care hospital has more than 1600 beds, and is equipped with all medical and surgical specialties. There are 19 outpatient departments (OPDs) and more than 5000 patients are served on a daily basis across the OPDs by 30 doctors in different departments; but the number of patients varies according to OPD. The patients can elect to see the doctor of their choice within each OPD and as a result the number of patients seen varies from doctor to doctor. The system by which the OPD operates involves obtaining roll call slips and then waiting in a queue for consultation by the doctor. Some medicines prescribed can be obtained from the hospital pharmacy, while patients have to purchase medicines from private sector pharmacies which are not available through the public sector. It is normal to dispense three days of medicines at hospital pharmacies. Collectively, the OPDs are served by a total of five pharmacies.

### Interview schema development and data collection

The interview schema was developed through formulating questions that answer the research problem and that address gaps in the literature. The interview schemas for each of the two cohorts are outlined in Appendix A. The patient interview schema contains 9 questions (with further sub-questions) covering the following broad areas: source of medicine-related information, reasons of unawareness about their dispensed medicines, consultation time, side effects of medicines, satisfaction with medicines information provided, benefits of patients having a copy of the prescription, and roles of pharmacist. The health professional interview schema was the same for the doctors and dispensers and contained 6 questions addressing the following broad areas: reasons for lack of patient knowledge about their dispensed medicines, system-related problems that serve as barriers in providing information to patients, doctors behaviour toward patients and importance of the pharmacist within the healthcare system.

Before conducting interviews with the recruited study participants, piloting of the draft interview schema was undertaken to test the interview protocols and ensure uniformity and face validity. This involved going through the interview in a mock fashion with a patient and a health professional to ensure that the questions were understood, as posed. This process took approximately 30 minutes per interview. The interview schema was modified and it resulted in a list of questions which were comprehensive and unambiguous.

Data were collected from May 2016 to July 2016. A purposeful sampling technique was applied to recruit both patients and health professionals [16]. The sample size was limited by applying the saturation point criteria [17].

Patients aged ≥18 years were approached consecutively outside the pharmacies and their consent to participate in the study was obtained after explaining the nature of the study to them. A checklist consisting of key indicators to assess the knowledge of the patients (about their dispensed medications) was developed for recruiting patients (Box 1) [18]. Patients were considered unaware of the use of dispensed medicines if they had insufficient knowledge about one or more indicators. Consented patients were interviewed face to face using the piloted interview guide in a comfortable place near the OPD to explore the reasons of their unawareness of the medicines they were taking.

**Box 1. Checklist consisting of key indicators to assess the knowledge of the patients about their dispensed medications**

**Checklist Parameters**

Purpose of therapy

Individual drug purpose

Dose/quantity

Frequency

When(at what time)

Route

Storage

Duration of therapy

Refill

There was a two stage process involving purposive and convenience sampling [19]. Registered medical doctors (those registered with the Pakistan Medical & Dental Council; PMDC) were purposively contacted by using the directory of OPD doctors provided by the administration of the BVH. Individual doctors were then recruited through a convenience sampling strategy based on their availability and willingness to participate and the interviews were conducted by personally visiting them in their OPD clinics. All adjoining pharmacies of the OPDs were also visited and the dispensers serving in them were interviewed following consent to participate.

The interviews were conducted in commonly used languages such as Urdu, Punjabi and Saraiki, in a comfortable and cordial environment. All interviews were recorded on an audio tape with permission (as part of the consenting process), and observation notes were also taken. The participants were offered the opportunity to listen to the recorded 1 or read the written interview transcript and amend as appropriate.

### Data analysis

This is a descriptive thematic study and the data were analyzed using an interpretative approach for analysing qualitative data [20]. The interviews were recorded and transcribed verbatim. The audio recordings of the interviews were listened to several times and the written transcripts were also read several times in-order to immerse oneself in the data to have a rich and deep understanding of it. The themes emerged following a series of coding steps. This is not a grounded theory study however, coding techniques most closely represented by a grounded theory like approach were adopted [21]. This is an acceptable approach to qualitative analysis and the process was undertaken as follows: Initial coding was completed by coding the entire transcript, while preserving the meaning of the participants’ words. Then axial coding was performed in which relationships were identified between the initial codes and they were grouped into categories. Lastly, selective coding was undertaken to organize the categories into themes and subthemes. Cross checking was undertaken by the research team at each step of the analysis to ensure data credibility and to enhance the trustworthiness of the data [20]. The coding was undertaken by MA and AS in tandem. The process involved AS undertaking the initial coding independently and then the research team meeting to challenge the themes that were emerging relative to the data and for them to be defended by AS. This is common qualitative research practice.

### Ethical considerations

Ethical approval was obtained from the Pharmacy Research Ethics Committee (PREC) at the Islamia University Bahawalpur (Reference: 44-2016/PREC, dated March 15, 2016). The purpose of the study was explained to all the participants (patients and healthcare professionals) prior to the interviews being conducted. Verbal consent was obtained from the participants which was audio recorded and this procedure was approved by the PREC Committee. Written consent was not possible in most cases because the majority of patients were illiterate and so verbal informed consent was the primary form. Explicit permission was obtained from the participants to publish the transcripts of their interviews.

To ensure confidentiality, both patients and healthcare professionals were assigned identifier numbers (e.g. participant 1, doctor 2, dispenser 3 etc.). The participants were given the choice to withdraw from the interview process at any time. All of the audio tape recordings were kept locked and secure and were not used in any other study. Password protected computers were used to store information throughout the duration of the study.

### Findings

A total of 19 patients and 16 healthcare professionals (10 doctors and six dispensers) were interviewed. Among the patients, 10 were males and nine were females, and among the healthcare providers, 10 were males and six were females. The average interview duration was 21.6 minutes (range 19 to 25 minutes; SD = 3.39) for the patients and 26.2 minutes (range 20 to 32 minutes; SD = 2.81) for the healthcare professionals, respectively. The age of the patients ranged from 20 to 77 years. Most of the patients were illiterate; suggesting they had no formal education. The characteristics of respondents are provided in Table 1.

**Table 1:** Characteristics of the respondents.

Respondent	Patients			Healthcare Professionals		
	Gender	Educational status	Interview duration (minutes)*	Gender	Specialization	Interview duration (minutes)*
01	Male	Illiterate	21	Female	Gynecologist	24
02	Male	Illiterate	20	Male	Eye Specialist	26
03	Female	Illiterate	19	Female	Gynecologist	32
04	Female	Illiterate	22	Female	Gynecologist	20
05	Female	Intermediate	19	Female	Endocrinologist	30
06	Male	Matriculation	25	Male	Eye Specialist	26
07	Male	Primary	25	Female	Skin Specialist	27
08	Male	Illiterate	23	Male	Child Specialist	26
09	Female	Illiterate	21	Male	Eye Specialist	24
10	Male	Illiterate	20	Female	Eye Specialist	25
11	Male	Primary	22	Male	Dispenser	25
12	Female	Illiterate	19	Male	Dispenser	23
13	Male	Primary	24	Male	Dispenser	27
14	Female	Tertiary	20	Male	Dispenser	29
15	Female	Illiterate	22	Male	Dispenser	29
16	Female	Illiterate	19	Male	Dispenser	27
17	Male	Primary	21			
18	Female	Illiterate	25			
19	Male	Matriculation	23			

*Rounded. Note: The term illiterate is used for respondents who had no formal education at all and could not read or write.

The data obtained from both groups of respondents (patients and healthcare professionals) were analyzed to understand the key factors affecting patient’s lack of knowledge about their dispensed medications. Through the data analysis process three major themes and 13 subthemes emerged. The emerged themes are outlined in Table 2 and description about the emergent themes and the supporting quotes make up the remainder of the Findings section.

**Table 2:** Themes, subthemes and categories of patients’ knowledge about dispensed medicines.

Themes	Sub themes	Categories	
		Patient’s perspective	Healthcare professional’s perspective
**Healthcare professional related factors (doctor/ dispenser)**	Behaviour and attitude	• The attitude of healthcare professionals was not good. • Healthcare professionals prioritized the referenced patients. • Healthcare professionals did not give due attention to the patients. • Patients were reluctant to ask about their medicines due to fear of insult.	• Healthcare professionals agreed that their behaviour was not good due to multiple reasons such as being overburdened, tired of checking patients continuously etc. • Patients were reluctant to ask about their medicines due to fear of insult.
	Professional liabilities	• Doctors did not educate the patients about the side effects of medicines. • Healthcare professionals did not provide adequate consultation time and so patients were not satisfied.	• Healthcare professionals admitted that since they did not provide adequate consultation time then patients were not satisfied.
	Liaison	• There was a lack of collaboration between doctors and dispensers.	• There should be collaboration between doctor and dispenser, but it was not possible due to a number of problems, mainly relating to the dearth of facilities and staff.
**Patient related factors**	Eagerness of the patients	• Patients had a desire to know about their medicines.	• Patients were keen and wanted to know about their medicines.
	Lack of understanding	• Improper understanding of medicine use, most probably due to language barrier.	• Lack of understanding of patients is due to illiteracy. • Language barrier is not an obstacle in providing the medicine related information to patients.
	Misconception	• Patients expected the medicines to show effects within three days. • Due to lack of knowledge, the patients may discontinue therapy if side effects appear.	• NA
**System related factors**	Patients with special needs	• Special labels should be used for illiterate patients.	• Specialized marking must be done on the medicine label for illiterate patients.
	Perceived role of the pharmacist	• Patients were unaware about the role of pharmacist. • Upon explaining the role of the pharmacist in a healthcare setting, patients agreed that pharmacists should be appointed.	• Pharmacists should be present in the healthcare system, which could be beneficial for both healthcare professionals and patients. Moreover, they can help in inventory management.
	Prescription and medicines	• Only three days of medicines were dispensed and patients were unaware about prescription refill. • Prescriptions were not given to patients.	• The majority of healthcare professional respondents stated that medicines should be given for more than three days. • Prescriptions should be given to the patients.
	Staff workload	• Number of healthcare professionals should be increased.	• Number of healthcare professionals should be increased, since the number of them is not proportional to the number of patients that need assessment and treatment.

NA = not applicable

### Healthcare professional (doctor/ dispenser) related factors

#### Behaviour and attitude

Most patients highlighted that the doctors were rude and arrogant. The patients were afraid to clarify any doubts they had or ask any additional questions of the doctors, due to fear of insult.

“……..I was nervous while talking to the doctor because I could see that the doctor was already angry.…….he was arrogant……..his behaviour was already so rough and rude that I did not have the courage and I did not ask due to fear of insult in front of so many people…………” (Patient no. 14)

The majority of the healthcare professionals reflected this patients perception that they did not behave in a good way toward the patients, most probably due to being overburdened and tired of checking patients nonstop from morning until evening. The pressure is also likely due to a lack of basic facilities available for life in general, as well as at the hospital. In addition, there is the perception that patients may have irritated doctors by asking questions repeatedly.

“…. I agree that our behavior is not good with the patients. The reason is that the doctors are overburdened. Doctors have to work non-stop in this system, so they also become tired and feel irritated. There are a number of patients at OPD and we cannot give sufficient time to a single patient. If we give them more time than we cannot see all the patients and this is the reason we behave rudely with the patients. …” (Doctor no. 10)

One doctor also emphasized that they should behave in a polite manner otherwise patients might not communicate properly with them, due to fear of being snubbed and this might ultimately have an impact on their treatment outcomes.

“….. Doctors should not behave rudely. If they do so, then patients become afraid of the doctors and do not tell their history properly. If you talk to them nicely then they will give you their medical details freely, without any hesitation…..” (Doctor no. 9)

Six patients were of the view that the patients with a reference from a doctor or member of society were given more time and attention and were exempt from the burden of waiting their turn to see the doctors. As a result, the rest of the patients had to wait longer. Most patients reported that patients without a reference were not given the attention they deserved.

“…………I have come here with a reference.… Nobody bothers about the person who does not have a reference………” (Patient no. 13)

Some patients said that the doctors were either busy with their friends or medical sales representatives, or were using their mobile phones. As a result, they were less concerned with the illness of patients.

“…. In fact I noted that the doctor is usually busy on his mobile…..Just carelessly asks what your problem is and says be quick, I do not have time….he did not even give me a minute, I think a few seconds. He just looked at me in a glance, listened to my problem and wrote the prescription while looking at his mobile and then called the next patient…….”(Patient no. 14)

The majority of healthcare professionals stated that they never indulged in using mobile phones or saw medical sales representatives and didn’t keep patients waiting as a result. Moreover, they noted that it was not written anywhere in hospital policy that the use of mobile phones was prohibited. The doctors interviewed suggested that they only used mobile phones when needing to make work-related emergency calls.

“…..No, this does not happen normally. Now if the doctor gets an urgent call, he might pick up the phone. There is no rule regarding this. The patient wants to get full attention. But the doctor might have to do some other things as well…..” (Doctor no. 7)

However, two healthcare professionals admitted that they used mobile phones or attended to discussions with medical sales representatives within the hours of duty. Despite this they gave due time to their patients.

“…..Yes, but it is the part of duty. Some phone calls are personal. Sometimes friends and relatives are coming. It is not mentioned in our duty rules that you cannot pick up the phone call and not meet your relatives and friends. But along with this, we give proper time to the patients and also dispense the medicines…..” (Dispenser no. 5)

#### Professional liabilities

While assessing the knowledge of the patients, we found that none were being educated about the possible hazards of their medicines. The patients were aware that medicines had a tendency to cause side effects. Most patients also reported that the consultation time given to each patient was minimal, ranging from a few seconds to a maximum of six minutes. As a result, most patients were confused about their medicines and dissatisfied about the service they had received.

“…..They just see the patient for a minute, ask them and say be quick we do not have time. They do not listen completely…..” (Patient no. 14)

Most of the healthcare professionals stated that they could not give proper consultation time to the patients due to being overburdened with the number of patients they had to see.

“….. We can give barely half a minute. I have sometimes seen 180 patients per day. You can calculate the time I can give to one patient…..” (Doctor no. 7)

Contrary to this, four healthcare professionals stated that they provided adequate consultation time to patients and did not allow them to leave unless they were completely satisfied that they had been dealt with. They further added that they always tried to provide a comprehensive explanation to patients about their medicines, as the majority of them were illiterate and it is pointless handing over the prescription to patients without proper explanation. Furthermore, these health professionals gave more time to the patients who came from rural areas some distance away or to the patients with chronic illness or serious acute ailments.

“….. Despite our busy schedule we give adequate time to patients. We counsel them properly and explain in detail about the medicine. Doctors are also aware of the fact that only handing over the prescription is useless for the patient because it is written in English language and patients are mostly illiterate, so we explain in detail. Even if the patient comes again with some ambiguity then we make it sure to clarify it…..” (Doctor no. 4)

#### Liaison

There was lack of concordance between the dispenser and the prescribing doctor. There was no system through which the dispenser could consult with a doctor or notify the doctor of an error in a prescription or suggest a change in therapy.

One patient was confused because the doctor had told the patient a different dose and frequency than what the dispenser was telling them. As there was no mechanism for liaison between the two, there was no way of clarifying any doubt. The only option was for patients to re-visit the doctor and go through the time-consuming process of getting slips and waiting in the queue again; which they were reluctant to do. This represents a fundamental deficiency in the system.

“….. The doctor told me to take one tablet a day while the dispenser told me to take two. So I discussed with the dispenser that I think that two tablets will be harmful, and as far as I remember the doctor prescribed only half tablet, twice a day. But the dispenser emphasized on taking two tablets a day, so I am confused. We come to hospital but are not satisfied….” (Patient no. 5)

Approximately half of the healthcare professionals aligned with the patients’ perception that there was a lack of collaboration between the doctor and dispenser. However, whether a mechanism for liaison exists or not, almost all of the healthcare professionals agreed that such collaboration is essential in-order for the system to run efficiently. In this way, the dispensers can clarify any medicines-related issues they may have with the doctors and the doctors would be more familiar with the medicines stocked by the pharmacy, so that they can make suggestions of alternatives in case particular medicines are unavailable. The doctors also stated that the absence of basic facilities such as telephones and shortage of appropriately trained staff were the main reasons for this lack of interdisciplinary integration and collaboration.

“….. We do not have sufficient staff. There are so many patients going from each OPD to the main pharmacy. There are other pharmacies as well but the majority are going to the main pharmacy. And there are only two to three people there to handle this crowd. The number of staff members is same as ten years ago. Two months ago I was attending 1700 patients alone. Then I complain to the authorities that I am unable to do this alone. How can there be collaboration in these circumstances?…..” (Doctor no. 5)

### Patient-related factors

#### Eagerness of the patients

While most of the patient participants were illiterate, almost all of them were eager to know about their medicines and their ailments in detail. They told us that there was no proactivity by the health professionals in terms of allowing questioning and that they had repeatedly tried to ask the doctor as well as the dispenser.

“…..I asked him and then he told me. I asked how I should take it. Usually they do not tell about the usage of medicines without asking…..” (Participant no. 7)

Some healthcare professionals agreed that patients were interested in knowing about their medicines and did not want to leave the hospital unless they were fully satisfied that they were informed. Since the doctors had to see a number of patients they were unable to answer all their queries.

“….. Patients are concerned. Patients are never in a hurry, however the doctors are…..” (Doctor no. 10)

#### Lack of understanding

Literacy levels of patients appeared to impact on the development of sound doctor-patient relations, resulting in improper and inadequate transfer of knowledge. One patient stated that it was very difficult for them to understand the instructions given by the doctor because of language differences.

“…..Yes he told me but I could not understand. I asked again how many times should I take the medicines, they replied just go away, do not disturb…….We are Saraiki people, so I did not understand……” (Patient no. 9)

The majority of healthcare professionals stated that illiteracy and access to technology were the major factors responsible for lack of knowledge among patients about their dispensed medicines. In contrast to this, educated patients generally possess sufficient knowledge about their medicines.

“…..The major reason is the patient’s illiteracy. However, lack of knowledge and lack of education are also responsible for patient unawareness. If some educated patient comes to us, then the moment we tell him the name of the medicine, they simply search for that medicine on the internet and get the information related to it…..”(Doctor no. 2)

Nearly all (14) healthcare professionals stated that differences in language had never been an obstacle to the transfer of medicines-related information. They always communicated with patients in their native language and later ensured that they correctly interpreted the meaning of their conversations.

“…. The patients should be told in simple words so that they understand…. Some people come here from Cholistan (desert area), some from other villages. They do not understand what we are saying. Then we ask them if they are Saraiki, Punjabi, Urdu or Muhajir. Then we try to make them understand in their own language. And after that we ask them to repeat what they have understood, to confirm…..” (Dispenser no. 4)

However, two healthcare professionals were of the view that language differences might be responsible for causing hindrance in the transfer of information, because the hospital staff are unlikely to be familiar with all the languages.

“….. Yes, language might be a barrier. We try to use our national language. Language must be simple and understandable. Patients who speak Pushto or other languages come as well. We do not know all the languages but we try to use the language that the patient understands and which satisfies him. But I agree that this does not happen always…..” (Doctor no. 8)

#### Misconceptions

The doctors did not inform most patients about the duration of therapy and the need to re-visit the hospital. This is in light of the fact that the dispenser only provides three days of medication to all patients. Upon asking the patients what they would do when their three day course is complete, the patients offered variable responses. Some patients said that they would come back to get a refill or a re-check-up if their disease is not cured and they would not come back if they thought they had recovered from their illness.

“…..No, I will not come again in case I recover. And in case I do not recover, I will come again…” (Patient no. 18)

On the contrary, other patients said that they would come back for a refill or re-check-up if they saw improvement in their condition.

“….Doctor did not tell about this. However, I will come back when these medicines will be finished. In case I start recovering, I shall come back….” (Patient no. 15)

A few patient respondents highlighted they would not come back and would visit some other doctor, if they have opted to use the three days of medication, because that means that the medicines were not working for them or that the doctor was not competent enough.

“….In case I recover I shall come back to hospital but if I did not recover I shall not come back….If these medicines are not beneficial then why should I use them…” (Patient no. 3)

None of the patients felt they were being educated about the side effects of the prescribed medicines. All the patients said that they would discontinue their therapy if any such effect occurred.

“….I shall not take medicines in case the side effects appear…..” (Patient no. 8)

### System-related factors

#### Patients with special needs

Upon asking what changes should be made to the system, three patients said that the dispenser should mark the medicines or put some kind of pictorial label on them so that illiterate patients can understand how and when to use them.

“……We are illiterate patients. They should carefully mark our medicines and tell us about frequency or if we should take it before or after meal. Then it would be beneficial for us….” (Patient no. 11)

The healthcare professionals agreed with the fact that the majority of the patients visiting the hospital were illiterate and there was a dire need to label the medicines appropriately. They explained that although the patients were given verbal information about their medicines, this was insufficient and it is likely patients would forget the instructions. Moreover, simply handing over the medication was not good practice either, because illiterate patients are not able to understand unless the medicines are labelled appropriately. They further added that the process of labelling was quite difficult in this setting, due to the overcrowding of patients and lack of staff.

“…… The system here is to tell verbally only. But actually we should mark the medicines. There should be separate small bags like at medical stores. And we should tell the patients that these are to be taken in the morning or evening or before or after breakfast. So that the patients can easily take out the medicines and see that these are for night or morning. They would not forget. But here there are neither such facilities nor time…..” (Dispenser no. 16)

Contrary to this, six healthcare professionals claimed that they labelled the medicines before dispensing, for facilitating patients’ medication taking.

“….. Yes, dispensers mark the medicines. For instance the eye drops are marked 2, indicating that these drops are supposed to be instilled twice daily…..” (Doctor no. 9)

#### Perceived role of the pharmacist

Unfortunately, none of the patients knew about the pharmacist role. They did not know that there is a qualified person who should be present at the pharmacies to educate them about dispensed medicines. However, upon explaining the role of the pharmacist to the patients (**Box 2**), all participants agreed that the pharmacist should be present at pharmacies to counsel them and that it would be of great benefit in assisting patient understanding and promoting their wellbeing.

“….Yes, there should be some good new profession, not like the existing one. They should tell the patient about the medicines and how to avoid from inappropriate medicine…….the patient would take correct medicine which would ultimately increase his life span….” (Patient no. 18)

**Box 2. Role of pharmacist being explained to the patients**

**Role of pharmacist**

- Provide medicine-related information
- Counsel the patient about medicine use
- Answer the queries of patients related to their medicines

Nearly all (15) of the healthcare professionals thought the presence of the pharmacist should be mandatory, for the betterment of the healthcare system. Since pharmacists possess medicine-related information, they are able to educate patients and help doctors by clarifying any medicine-related issues. This would be of prime importance in reducing the burden placed on doctors regarding medicines. Moreover, pharmacists could also help to maintain the inventory of medicines and to keep checks and balances on the quality of medicines in the hospital.

“….. There should be a pharmacist in the hospital. He can coordinate with the doctor. Dispenser will run the dispensary and pharmacist will deal with the routine problems like shortage of medicines. His presence will benefit the patients as well as the healthcare professionals. Our burden will be reduced. The pharmacist can discuss with the doctor if there is a problem and will also put efforts to make the medicines available…..” (Dispenser no. 4)

#### Prescription and medicines

Only three days of medication is routinely dispensed and almost all patients were not told about refills. One patient stated that since he was a chronic patient, he should have been given medicines sufficient to cover two weeks, since it was difficult for him to re-visit the hospital.

“……I am a cardiac patient and they just gave me medicines for three days. They should have given the medicines for at least 15 days. (Patient no. 1)

Surprisingly, we found a range of responses when interviewing healthcare professionals about dispensing of three days of medicine. Six healthcare professionals suggest that although it was hospital policy to provide only three days of medicine, still they prescribed and dispensed medicine for more days because they believe that three days of medicines is insufficient to meet the needs of most patients.

“….. We prescribe medicines for at least one week. I believe that giving only three day medicine to the patient is not sufficient and this system should be changed. For instance, if it is required for a patient to take calcium tablets for a month, then it is useless to give him three day medicine only. He should be given medicine for the entire duration i.e. 30 days…..” (Doctor no. 10)

The majority of healthcare professionals agreed that medicines should be prescribed for more than three days but unfortunately this practice was not prevalent in the hospital due to the policy restriction.

“….. I believe, if a patient is prescribed antifungals for his problem, and he has to use those medicines for 4 weeks or has to take 4 tablets, and we give him 2, he would not be able to complete his treatment and cause problem for the patient. So the medicines should be dispensed in sufficient number required to complete the treatment……” (Dispenser no. 4)

Contrary to this, some healthcare professionals were of the opinion that the current system of giving three days of medicine was good. They were of the view that the general population is not aware of the harmful effects of medicines, so in order to avoid overdosing, three days of medicine is enough. Moreover, the patients should come back after three days so that their dose could be tapered according to their condition at that time.

“….. I think this system is completely fine. Medicine usage is a sensitive issue. These are not mere sweets and candies which can be consumed as much as you want. We give medicines for three days and ask the patient to come back for follow up. When he comes back, we examine him and taper the dose accordingly…. We have to monitor the dose and side effects as well. Medicines should not be given for more than three days at any cost. Patient has no idea but that medicine may be lethal for him…..” (Doctor no. 4)

The healthcare professionals also said that since BVH is a resource-constrained setting then providing all patients with lengthy supplies of medication would exhaust resources further and result in significant shortages.

“….. Hospital has a limited number of medicines. If we give 15 day medicine to a patient then the medicines will run out of the stock. So, in order to provide medicines to all the patients, three day medicine is given. This is due to lack of budget…..” (Dispenser no. 3)

All the patients reported that their prescriptions were kept at the pharmacy for maintaining records. The patients said that they should be provided a carbon copy of the prescription to follow the treatment plan. Some patients said that they should have the prescription for keeping a record of which medicines they have used and for showing it to the doctor on the next visit and it would also facilitate patients purchasing the medicines from their own districts or villages that they return to following the hospital visit.

“…If I would have the prescription, I could tell the doctor which medicine I have taken if my condition worsens…If I do not have the prescription, the doctor would have to re-evaluate on next visit….” (Patient no. 10)

According to the healthcare professionals, prescriptions were only given to the emergency or chronic patients such as those in coronary care units or psychiatric wards. They agreed that a copy of a prescription must be handed over to the patients so that they can show it to the doctor on their subsequent visit to the hospital and that it would also help in case the patients forget about their medicine. There were two major reasons for not implementing this practice; first, the hospital staff had to keep the prescription with them for the sake of audit; second, there was overcrowding of patients and a shortage of hospital staff. The hospital staff were already overburdened with work, caring for patients and they do not have ample time to make carbon copies of prescriptions and hand them over to patients.

“….. Prescription should be given to patients….. There should be a system in which a copy of prescription should be handed over to the patients. In this way, one copy could be given the patient and the original can be used for audit. In this way the patient can show his medication record to the doctor on subsequent visits…..” (Doctor no. 9)

#### Staff workload

Patients suggested that the number of healthcare providers should be increased to allow proper consultation and dispensing times. A few patients said that the number of both doctors and dispensers should be increased while most said that the number of dispensers was adequate, but that there should be more doctors.

“…..If the number of doctors would increase, then the patients would not have to wait. Right now, one doctor has to attend 200 to 300 patients. The whole room is filled….The dispensers are enough and they dispense quickly….” (Patient no. 7)

The healthcare professionals also agreed with the perspective of the patients, that due to shortage of staff they could not give proper time and attention and as a result, patients might be neglected at times. They said that it was the responsibility of the hospital authorities to appoint more staff i.e., both the doctors and dispensers for the wellbeing of the patients.

“….. Doctors are extremely overburdened and so they could not give proper time to the patients….. If you want to examine the patient honestly, you need 30 minutes or even more. After 30 minutes you can make provisional diagnosis. For proper examination, at least 30 minutes are required. In this hospital we have to examine 500 patients and if we divide the time according to the number of patients then we cannot even give 30 seconds to a single patient…..” (Doctor no. 2)

A brief summary of the factors affecting the patient’s knowledge about dispensed medicines is given in Box 3.

**Box 3. Factors affecting patient’s knowledge**

- Rude behaviour of healthcare professionals
- Inadequate attention and time by healthcare professionals
- Hesitation among patients in asking about medicines due to fear of insult
- Insufficient education provided by healthcare professionals
- Language barrier between healthcare professionals and patients
- Illiteracy of patients
- No specialized marking (labelling) on medicines for illiterate patients
- Unavailability of pharmacists
- Copy of prescriptions not provided to patients
- Healthcare professionals are overburdened

## Discussion

Our qualitative study aimed to explore the factors affecting patients’ knowledge about dispensed medicines. What emerged from data analysis was 31 categories (patients = 19, healthcare professionals = 12), 10 subthemes and 3 themes. The themes encompass healthcare professional-related factors, patient-related factors and system-related factors. In the first part of this study, questions were asked from the patients in-order to identify the factors responsible for their inadequate medicine related information. In the second part healthcare professionals were asked about their views. Their perspective was equally important and it helped to answer most of the questions raised in the first part of the study.

### Healthcare professional-related factors

The information provided by the doctors is more likely to be dependent on their attitudes than on their knowledge [22]. It is quite obvious that the doctors basic relational attitudes i.e., being friendly, avoiding being judgmental and taking interest in the patients [23] have a very positive impact on the patient. Our study at BVH clearly indicated that the doctors’ attitude was not good toward patients, and both the dispensers and the doctors were not giving patients due attention. The healthcare professionals also mostly agreed with the patient’s perspective that they did not behave in a professional manner toward them, largely due to the excessive overcrowding of hospitals, the robotic life style i.e., working from morning till evening without a break until late afternoon, and the repetitive questions from patients that needed to be answered. This is in line with the findings of other studies where doctors are reported to be extremely overburdened and unable to spend the appropriate duration of time dealing with patients, leading to compromised work quality [24–26]. The current study showed that those patients who came with a reference were prioritized by the doctor; as a result, they did not have to wait for their turn in a long queue and were given more time and greater attention. A study conducted in San Francisco, USA reported that these sorts of disparities raise concerns in the minds of people about the potential for bias by clinicians and administration/leadership [27]. Furthermore, patients in this study wanted to ask the doctor about various medicine-related ambiguities but fear of being snubbed by health professionals reduced their confidence in doing so. This is similar to findings of a study conducted in the United Kingdom [28]. The doctors in our study confessed that they should be polite with the patients so that two-way communication can be established. It is expected that this friendly environment will facilitate better patient outcomes both in terms of their health and their satisfaction with the system [29].

Knowledge about potential side effects of a medicine is helpful for patients in order to recognize side effects early, and promptly report these to medical practitioners. A Canadian study indicated that experiencing side effects often results in discontinuation of therapy [30]. Findings from our study support this notion, where the doctors did not educate patients about the side effects of medicines and so the patients even considered minor side effects as potentially significant. This meant patients were ready to quit the medicines at the first sign of a side effect. A study conducted in North India also reported that the provision of appropriate medicines-related information, including information pertaining to side effects is crucial to ensuring continuation of drug therapy [31]. A Spanish study reports that if adequate consultation time and appropriate information is actively given to patients then the risk of stopping medicines due to side effects can be significantly decreased [32]. In this manner, appropriate consultation time may not only prevent harmful consequences to health, but may also increase patient satisfaction with health professionals and the system in general. The findings of our study indicate that the doctors did not give adequate consultation time to the patients, ultimately leading to their dissatisfaction. Multiple Pakistani studies have also reported similar fact where doctors did not give ample consultation time to the patients [13, 14].

Furthermore, no alignment was seen between the doctors and dispensers and the absence of such liaison precludes the dispenser from discussing any errors in the prescriptions or any other medicines-related matters. The hospital staff admitted that such collaboration is vital for avoiding confusion, but the lack of basic facilities and shortage of staff were the major hindrance in this regard. An Irish study suggested the same, that proper communication between healthcare professionals regarding patient’s medicines leads to significantly improved patient care [33].

### Patient-related factors

In this current study, the patients were keen to know about their medicines so they asked the doctors and dispensers frequently about this. This indicates a desire and the positive attitude of Pakistani patients which might contribute to better disease management in an otherwise stretched health care system. This finding supports the literature from the developed world which suggests patients are often not provided with sufficient medicines-related information and generally want to learn more about their medications [34].

A study conducted in Gambia, Western Africa demonstrated that another factor which is frequently taken for granted while addressing the quality of dispensing instructions in a busy pharmacy is the language barrier; most likely in those settings where numerous languages are used for communication, but the dispenser has command of only a few of them. [6]. According to our findings, patients were unable to follow the instructions of both doctor and dispenser, largely due to language barriers. This aligns with the findings of several other relevant studies [35–37]. A South African study has shown that differences in language may lead to poor diagnosis and follow up, reduced quality of patient care and adherence to medical advice [38]. The data from our study suggests that BVH staff believe there is a blame culture surrounding the health professionals and the reality may be different. The health professional cohort in this study expressed that they always communicate with patients in the language which is understandable by them.

A common misconception which emerged in our study was the assumption by the patients that medicines generally produce their effects within three days and if the effect was not seen within this duration then there was no benefit in using that particular medicine. Moreover, patients in this study had no idea about the side effects of the medicines or the level of severity of these possible effects. The flow on effect of this is that it reduces the credibility of the doctors in the eyes of the patient because the patient believes that if a medicine is not showing effects in a short period of time, or is causing harm, then it is the doctor’s fault. If the patients were being educated about the side effects of the medicines, they would recognize and report them, rather than blaming the doctor and supposedly this would improve doctor-patient relationships.

### System-related factors

According to a study conducted in Sri Lanka, it is important that patients should at a minimum, receive well-labelled medications, and should understand their medicines and the associated side effects, to ensure optimal adherence [39]. The findings of our study indicated that since the majority of patients were illiterate they were unable to understand the instructions outlined on medication labels. The patients stated that specialized marking or symbols should be used by dispensers to allow illiterate patients to understand them. The dispensers also agreed with the patient’s perspective but they believed that patient overcrowding was the main limitation, through minimal time being spent on labelling. A study conducted by Dowse et al. reports that the addition of pictograms to medicine labels was associated with substantial improvement in understanding the instructions regarding administration which led to better patient adherence [40].

Pharmacy nowadays is moving towards more intervention oriented practice [41]. Pharmacists working collaboratively with patients can aid in maximizing clinical benefits and decreasing negative health outcomes. Interaction with the pharmacist affects patient’s expectations and increases their satisfaction with the health services provided [42]. The findings of our study suggest that patients are unaware of the presence of pharmacists and their roles and responsibilities. However, upon explaining the role of the pharmacist (Box 2) they agreed that such a health professional is vital for the healthcare system and there is a need to appoint pharmacists within the hospital setting for the benefit of patients. This lack of awareness of the pharmacist and their utility as a medicines expert is not limited to developing countries [43]. It is heartening that the healthcare professional cohort in this study also highlighted the importance of the pharmacist. They consider pharmacists to be the most suitable professional to manage medicines-related information and that the presence of the pharmacist is seen as beneficial not only for the patients but for the doctors and dispensers as well. A study conducted in Jordan also reported similar perceptions of doctors about the roles of hospital pharmacists [44].

In our study, another problem leading to impaired knowledge of patients about their medicines was the unavailability of a copy of the prescription to take to another pharmacy, post the hospital visit. Both patients and healthcare professionals in our study were of the opinion that at least a carbon copy of the prescription must be given to the patients; however, prescriptions were kept at the dispensary for the sake of maintaining records and no duplicate copy was handed over to the patients. This is associated with significant drawbacks, for instance if the prescription is not returned to the patients they may not be able to recall the medicine related information; moreover, if the patients run short of their medicines then they will not be able to purchase that medicine from private sector pharmacies. Having a copy of the prescription could also enable patients to show it to the doctor on the next visit. A Swedish study found that giving the original prescriptions to the patients and keeping their copy at the pharmacy leads to increased medicine adherence [1].

The findings of our study suggest that only three days of medication were dispensed to the majority of patients, despite having both acute and/or chronic ailments. Patients were not informed of the need or otherwise to refill prescriptions. Consequently, patients seemed to be largely unaware of the need to undertake a second visit. These are systems-based anomalies and a likely result of there being no integrated primary and secondary health care sectors in Pakistan. The health system needs to be strengthened to lay a foundation in-order for other parts of the sector to work, such as the pharmaceutical system [45, 46]. Healthcare professionals in the current study agree with the patient’s perspective that it would be most helpful to provide a reasonable quantity of ongoing medicines for chronic diseases and the entire duration of therapy for acute illness in a single dispensing.

The limitations of this study include the fact that the majority of the patients enrolled were illiterate and come from poor socioeconomic backgrounds; therefore, factors relevant to educated patients and those living in urban areas could have been missed. In some respects, this is the contribution of this paper; a focus on medicines-related issues in a provincial hospital in the developing nation of Pakistan. With this in mind the findings cannot be generalized to the entire country or abroad, because of differences in the health care system and basic facilities available in different hospitals.

## Conclusions and recommendations

Our qualitative study explored the important factors affecting patient’s knowledge about dispensed medicines from a hospital in Bahawalpur, Pakistan. Patients emphasized that professional behaviour, good conduct and attitude of healthcare professionals can have a very positive effect on them as patients. There is a need to establish collaboration between the doctor and dispenser at BVH so that any medicines-related issues can be clarified and any doubts about individual patients and their medications can be resolved.

Certain factors such as illiteracy, misconceptions and poor financial conditions serve as major hurdles in patients’ acquisition of knowledge about their medicines. Patients concerns about their ailments and associated medicines provide a very positive sign and such eagerness may lead to increased levels of medicine adherence. Pharmacists can bring a remarkable change by imparting medicine-related knowledge to patients and there is a strong need to make appointments of pharmacists within hospitals in Pakistan. Providing a duplicate copy of prescriptions to patients and informing them of the need to obtain a prescription refill (or not) should lead to a better understanding of their medicines, thereby improving adherence. The number of doctors and dispensers is not proportionate to the number of patients and so there is a requirement for more staff to be employed at the hospital under study. If these systems-related problems are addressed then the number of issues faced by patients can be significantly reduced, resulting in positive impact on medicines-related knowledge of patients from rural Pakistan.

## Supporting information

S1 Text COREQ checklist. (DOCX)
