# Supplementary material for: Factors affecting patients’ knowledge about dispensed medicines: A Qualitative study of healthcare professionals and patients in Pakistan
Source: PLoS One. 2018 Jun 1;13(6):e0197482. doi: 10.1371/journal.pone.0197482 (PMC5983558; doi:10.1371/journal.pone.0197482)
Supplement: S1 Text — (DOCX) [file pone.0197482.s001.docx]

| **Topic** | | **Item No.** | | | **Guide Questions/Description** | **Answers** | **Reported on Page no.** |
| --- | --- | --- | --- | --- | --- | --- | --- |
| **Domain 1: Research team and reflexivity** | | | | | | | |
| *Personal characteristics* | | | | | | | |
| Interviewer/facilitator | 1 | | | Which author/s conducted the interview or focus group? | | AS,RI,FR,MAB | 38 |
| Credentials | 2 | | | What were the researcher’s credentials? E.g. PhD, MD | | Pharm D | - |
| Occupation | 3 | | | What was their occupation at the time of the study? | | Students | - |
| Gender | 4 | | | Was the researcher male or female? | | Female/Male | - |
| Experience and training | 5 | | | What experience or training did the researcher have? | | Attended a workshop on methods of conducting qualitative study | - |
| *Relationship with participants* | | | | | | | |
| Relationship established | 6 | | | Was a relationship established prior to study commencement? | | Before starting the interviews, a friendly relationship was established with the consented participants | 10-12 |
| Participant knowledge of the interviewer | 7 | | | What did the participants know about the researcher? e.g. personal goals, reasons for doing the research | | Reasons for doing the research in-order to suggest changes in Pakistani healthcare system | 10 |
| Interviewer characteristics | 8 | | | What characteristics were reported about the inter viewer/facilitator? e.g. Bias, assumptions, reasons and interests in the research topic | | Reasons and interest in research topic | 10 |
| **Domain 2: Study design** |  | | | | | | |
| *Theoretical framework* | | | | | |  |  |
| Methodological orientation and Theory | 9 | | | What methodological orientation was stated to underpin the study? e.g. grounded theory, discourse analysis, ethnography, phenomenology, content analysis | | Inductive thematic analysis | 11 |
| *Participant selection* | | | | | | | |
| Sampling | 10 | | | How were participants selected? e.g. purposive, convenience, consecutive, snowball | | Purposeful sampling technique was applied to recruit both types of study participants | 9 |
| Method of approach | 11 | | | How were participants approached? e.g. face-to-face, telephone, mail, email | | Face-to-face interviews | 10 |
| Sample size | 12 | | | How many participants were in the study? | | 19 patients+16 healthcare providers | 12 |
| Non-participation | 13 | | | How many people refused to participate or dropped out? Reasons? | | 14 patients and three doctors refused to participate because either they were in a hurry or not willing to participate in the study. | - |
| *Setting* | | | | | | | |
| Setting of data collection | 14 | | Where was the data collected? e.g. home, clinic, workplace | | | Workplace (Hospital) | 8 |
| Presence of non-participants | 15 | | Was anyone else present besides the participants and researchers? | | | Moderator | - |
| Description of sample | 16 | | What are the important characteristics of the sample? e.g. demographic data, date | | | Patients (10 male,9 female), Healthcare professional (10 male,6 female) | 12, Table 1 |
| *Data collection* | | | | | | | |
| Interview guide | 17 | | Were questions, prompts, guides provided by the authors? Was it pilot tested? | | | Yes prompts were provided by the researchers. Piloting was done. | 9 |
| Repeat interviews | 18 | | Were repeat interviews carried out? If yes, how many? | | | There were no repeat interviews. However, if case further clarifications were required from the consented participants, they were contacted through a phone call | - |
| Audio/visual recording | 19 | | Did the research use audio or visual recording to collect the data? | | | Audio recording | 10 |
| Field notes | 20 | | Were field notes made during and/or after the interview or focus group? | | | Yes | 11 |
| Duration | 21 | | What was the duration of the interviews or focus group? | | | Patients (mean = 21.6 minutes), Healthcare professionals (mean = 26.2 minutes) | 12 |
| Data saturation | 22 | | Was data saturation discussed? | | | Yes | 9 |
| Transcripts returned | 23 | | Were transcripts returned to participants for comment and/or correction? | | | Returned only to the participants who demanded for it | - |
| **Domain 3: analysis and findings** | | | | | | | |
| *Data analysis* | | | | | | | |
| Number of data coders | 24 | | How many data coders coded the data? | | | 5 | - |
| Description of the coding tree | 25 | | Did authors provide a description of the coding tree? | | | Yes | 11 |
| Derivation of themes | 26 | | Were themes identified in advance or derived from the data? | | | Themes, subthemes and categories were derived from the data | 13 |
| Software | 27 | | What software, if applicable, was used to manage the data? | | | None | - |
| Participant checking | 28 | | Did participants provide feedback on the findings? | | | They were not contacted | - |
| *Reporting* | | | | | | | |
| Quotations presented | 29 | | Were participant quotations presented to illustrate the themes/findings? Was each quotation identified? e.g. participant number | | | Yes | 13 |
| Data and findings consistent | 30 | | Was there consistency between the data presented and the findings? | | | Yes | 13 |
| Clarity of major themes | 31 | | Were major themes clearly presented in the findings? | | | Yes | 13, Table 2 |
| Clarity of minor themes | 32 | | Is there a description of diverse cases or discussion of minor themes? | | | Both | Table 2  Refer to quotes in result section |
